# Smartphone Addiction, Use Preferences, and Depression Among Older Adults in the Digital Context: Machine Learning Analysis of Survey Data

**DOI:** 10.2196/84703

**Published:** 2026-03-12

**Authors:** Sheng Chen, Yue Song, Chien-chung Huang

**Affiliations:** 1 School of Public Administration, Guangdong University of Foreign Studies, 2 Baiyun Avenue, Baiyun District, Guangzhou, 510420, China, +86 13306695767; 2 School of Social Work, Rutgers University, New Brunswick, NJ, United States

**Keywords:** depression, fuzzy-set qualitative comparative analysis, machine learning, smartphone addiction, smartphone use preference

## Abstract

**Background:**

The increasing use of smartphones among older adults offers new opportunities for social connection but may also pose risks associated with adverse mental health outcomes, including depression.

**Objective:**

This study examined the relationship between smartphone use and depression among older adults in Guangzhou, China, to identify key predictors and complex configurations associated with depression.

**Methods:**

Using a hybrid analytic approach applied to survey data from 2585 older adults in Guangzhou, machine learning methods first identified the strongest predictors of depression. Subsequent fuzzy-set qualitative comparative analysis delineated distinct configurations associated with depression.

**Results:**

The analysis identified high levels of smartphone addiction and a low preference for interactive use as central configurations associated with depression. These patterns appeared to operate through 2 distinct mechanisms: structural exclusion related to resource limitations and agentic maladaptation associated with relational deficiencies. This underlying vulnerability, in combination with specific sociodemographic factors, delineated distinct depression-related typologies. High-risk profiles included unmarried men with limited educational attainment and socially withdrawn individuals with greater socioeconomic resources.

**Conclusions:**

Problematic smartphone use, particularly when it displaces social interaction, is significantly associated with depression among older adults. Interventions should therefore promote healthier interactive digital behaviors and provide targeted support for high-risk groups.

## Introduction

The rapid development of digital technologies, coupled with an aging population, has resulted in increasing smartphone use among older adults. The proportion of individuals aged 60 years and older in China increased from 14.9% in 2013 to 21.1% in 2023, reflecting the country’s transition toward a moderately aging society [1]. According to the 56th Statistical Report on China’s Internet Development, released in July 2025, the internet penetration rate among older adults in China reached 52%, with 161 million internet users aged 60 years and older [2]. These trends reflect the growing digital engagement of older adults in contemporary society.

The expanding prevalence of smartphone use has generated increasing scholarly interest in its implications for the mental health of older adults. Studies suggest that smartphones fulfill daily needs for communication, entertainment, and information [3]. However, problematic smartphone use has been associated with reduced social connectedness, intergenerational tension, and sleep disturbances [4], all of which may contribute to psychological distress. Thus, smartphone use presents both potential benefits and risks for older adults: moderate and purposeful engagement may support well-being, whereas excessive or maladaptive use may exacerbate mental health risks.

Despite a growing body of research, many studies on late-life depression tend to examine physiological, psychological, or social predictors in isolation, often underemphasizing the complex interaction among these dimensions and their combined effects. The limitation of single-factor studies constrains understanding of how digital behaviors, social contexts, and individual vulnerabilities interact to produce divergent outcomes. The influence of smartphone use intensity and preference does not occur in isolation but is embedded within broader life circumstances. To address this theoretical and methodological gap, this study developed a configurational model to identify the most salient predictors and examine how these factors combined to form patterns associated with depressive outcomes. The findings provide empirically grounded insights to inform strategies aimed at promoting mental well-being and digital literacy among older adults.

### Depression in Later Life

Late-life depression is a multifactorial condition influenced by physiological, psychological, and social factors. Physiological contributors include chronic illness and functional decline [5,6]. Psychological factors such as negative thinking and loneliness are also associated with increased risk [7,8]. Social support systems and community engagement play important roles. Social participation theory suggests that engagement in activities such as volunteering and recreational activities protects mental health by reducing loneliness and building supportive networks [9,10].

### Smartphone Use and Depression Among Older Adults

In this study, smartphone addiction is defined as a behavioral addiction characterized by compulsive and excessive use that disrupts daily functioning and is associated with adverse psychological outcomes [11,12]. Smartphone addiction has been positively associated with depression, particularly with prolonged use [13]. Excessive reliance may create a cycle in which loneliness drives escapist use, further deepening social isolation [14].

Patterns of smartphone use are associated with mental health outcomes. Purposeful and socially oriented use—categorized as interactive functions (eg, social networking and online task engagement)—may enhance social connectedness [15]. In contrast, entertainment-driven use—categorized as self-entertainment functions (eg, playing games, scrolling social media, watching videos)—is linked to higher levels of depression and anxiety, often mediated by escapism [16,17]. Although much of the evidence derives from studies of younger populations [18-20], older adults may be particularly vulnerable due to cognitive decline, which may increase the likelihood of passive use reinforcing isolation and depressive symptoms.

### Conceptual Framework

The conceptual framework of this study integrates socioemotional selectivity theory (SST) and the digital inequality theory (DIT). Socioemotional selectivity theory posits that older adults prioritize emotionally meaningful relationships, thus smartphone use may be beneficial only when it supports these goals rather than displaces them [21]. Digital inequality theory emphasizes that structural disadvantages limit digital literacy and access, potentially transforming technology into a source of exclusion rather than connection [22]. Together, these perspectives inform the hypotheses in this study regarding the conditions under which depression may arise among older adults. Accordingly, this study tested the hypothesis that depression among digitally active older adults is associated with maladaptive patterns of smartphone use. It further examined whether socioeconomic barriers limit beneficial engagement, contributing to exclusion and depressive symptoms, consistent with DIT, and whether individuals with greater socioeconomic resources substitute smartphone use for meaningful social participation, potentially resulting in emotional dissatisfaction despite material security, consistent with SST.

This study tested 3 hypotheses. First, high smartphone addiction was hypothesized to represent a necessary condition for high levels of depression. Second, high smartphone addiction in combination with low educational attainment and low preference for interactive use was hypothesized to constitute a sufficient configuration for depression. Third, high smartphone addiction combined with low social participation was hypothesized to form a sufficient configuration for depression, particularly among individuals with greater socioeconomic resources.

## Methods

### Data

Data were collected using a combined convenience and quota sampling strategy through a study-specific survey titled Smartphone Use and Mental Health Among Older Adults, administered between October and November 2024. To ensure data quality, structured, on-site assisted data collection was implemented across 87 social workstations in 5 districts of Guangzhou. Each workstation recruited 30 community-dwelling older adults. Social workers organized in-person sessions at community centers, guided participants through the questionnaires, clarified items, assisted with smartphone use, and reviewed responses for completeness. This assisted approach helped address technical and cognitive barriers, resulting in a high valid response rate and a consistent sample size.

### Measures

Depression was measured using the 15-item Geriatric Depression Scale, adapted by Tang [23] for Chinese older adults. Items were answered in binary form (yes=1, no=0), yielding total scores ranging from 0 to 15. Following Tang’s guidelines, scores were categorized as 0 to 4 (no depression), 5 to 9 (mild depression), and 10 points or more (clinical depression). Internal consistency was good (Cronbach α=0.790). Smartphone addiction was assessed using the 17-item Mobile Phone Addiction Index [24]. Items were rated on a 5-point Likert scale, with higher scores reflecting greater severity. In this study, the scale scores were summed and standardized. Reliability in this sample was excellent (Cronbach α=0.968).

Smartphone use preferences were assessed with 2 multiple-choice items: 1 captured the general smartphone functions used and another identified the 3 smartphone functions most frequently used. Exploratory factor analysis of 12 functions yielded three factors: (1) self-entertainment function use (eg, streaming and music; explained variance=3.627), (2) interactive function use (eg, WeChat, short videos; explained variance=1.264), and (3) basic telecommunication (eg, calls, texts; explained variance=1.122). Short video platforms, such as TikTok and Xiaohongshu, play an increasingly prominent role in fostering social connectivity and interaction. These platforms enable users to share videos with family members and engage in interactive livestreams within local community contexts [25,26]. Self-entertainment use and interactive use were the 2 functions retained for analysis. The use of self-entertainment or interactive functions was operationalized as the occurrence of engagement with self-entertainment or interactive features, with higher counts indicating stronger preferences.

Social participation was measured using the 22-item scale developed by Ren et al [27]. Responses were recorded on a 5-point Likert scale, with higher scores indicating greater participation. In this study, scores were summed and standardized. Internal consistency was high (Cronbach α=0.946). Covariates included gender, age, education, employment, self-rated health, income, marital status, and average daily smartphone use. Multicollinearity diagnostics indicated no concerns (all variance inflation factors <2).

### Methodological Approach

We used a multimethod strategy that combined machine learning and fuzzy-set qualitative comparative analysis (fsQCA). The integration of machine learning and fsQCA followed a predictive-explanatory sequence. Machine learning was used to handle high-dimensional feature selection and objectively identify factors most relevant for prediction, whereas fsQCA was used to uncover how these factors combine to produce depressive outcomes. This sequential design leveraged the strengths of both methods: machine learning identified the most influential predictors of depression, and fsQCA revealed complex configurations underlying depressive outcomes. Together, these provided a more nuanced understanding of complex relationships.

First, 4 machine learning algorithms—logistic regression, support vector machine, random forest (RF), and extreme gradient boosting—were applied using the web-based Python Jupyter Notebook development environment to identify salient predictors of depression. To prevent overfitting, 10-fold cross-validation was used, and performance metrics were reported as the mean across folds to provide stable and generalizable estimates. To enhance interpretability, the Shapley additive explanations algorithm was used, quantifying each variable’s contribution to model predictions.

Following predictive modeling, fsQCA was conducted to test hypotheses on the necessary and sufficient conditions for depression. Fuzzy-set qualitative comparative analysis is a set-theoretic method that evaluates causal complexity by identifying configurations of factors, rather than the net effects of individual variables [28]. Based on fuzzy-set theory, it assesses necessary conditions, sufficient pathways, and conjunctural causation, allowing for equifinality (ie, multiple combinations of conditions leading to the same outcome). This approach is well-suited for exploring how structural vulnerabilities, smartphone use patterns, and social participation jointly shape mental health in later life.

### Ethical Considerations

This study was conducted in accordance with the Declaration of Helsinki and approved by the Research Review Committee of the School of Public Administration, Guangdong University of Foreign Studies (2024003). Informed consent was obtained prior to survey administration. Participants were informed that participation was voluntary and anonymous, that they could withdraw at any time, and that measures were in place to ensure data confidentiality, such as deidentification, secure server storage, and restricted access to data.

## Results

Of 2610 questionnaires distributed, 2585 were completed and deemed valid, yielding a 99% response rate. Demographic characteristics are presented in Table 1.

**Table 1.** Statistics of demographic characteristics (N=2585).

Characteristics	Participants, n (%)
Depression	
1‐4 points, no depression	1429 (55.3)
5‐9 points, mild depression	983 (38.0)
10 points and above, clinical depression	173 (6.7)
Use interactive functions	
No	387 (15.0)
Yes	2198 (85.0)
Use self-entertainment functions	
No	2056 (79.5)
Yes	529 (20.5)
Gender	
Female	1719 (66.5)
Male	866 (33.5)
Age (years)	
60-69	1490 (57.6)
70-79	729 (28.2)
≥80	366 (14.2)
Education level	
Primary school or below	1056 (40.9)
Junior or senior high school	1205 (46.6)
College and above	324 (12.5)
Employed	
No	2337 (90.4)
Yes	248 (9.6)
Health status	
Unhealthy	1935 (74.9)
Healthy	650 (25.1)
Monthly income^a^	
Less than 2000 RMB	797 (30.8)
2000‐5000 RMB	1224 (47.4)
More than 5000 RMB	564 (21.8)
Marital status	
Single	971 (37.6)
Married	1614 (62.4)
Smartphone use duration	
1 hour or less	520 (20.1)
2‐6 hours	1711 (66.2)
7 hours or more	354 (13.7)

a A currency exchange rate of 6.9 RMB=US $1 is applicable.

### Predictive Analysis Using Machine Learning Models

A comparative analysis was conducted using logistic regression, support vector machine, RF, and extreme gradient boosting models to predict depression based on smartphone use variables, social participation, and sociodemographic covariates. The dataset was partitioned into a training set comprising 80% of the data and a test set comprising the remaining 20%. During feature engineering, the outcome variable was binarized and the predictor variables were standardized. This process transformed all features to a common scale; this ensured their influence was balanced and prevented certain features from having an excessive impact on the model due to differences in magnitude. To reduce the risk of model overfitting, bootstrapping was applied within the machine learning framework to generate training sets for each tree, resulting in 1740 additional resampled observations, which were used exclusively for model training; all subsequent analyses were conducted using the original dataset.

To address observed imbalances in the outcome variable (depression categories) within the training data, the synthetic minority over-sampling technique was applied to the training set only and not to the test set used for final area under the curve computation. After applying the synthetic minority over-sampling technique, the distribution of depression levels was fully balanced, providing a more equitable foundation for model training. Grid search with 10-fold cross-validation was used for hyperparameter tuning and model optimization. Among the 4 machine learning analyses conducted, the RF model demonstrated superior performance on the test set as shown in Table 2; therefore, RF was selected for feature importance analysis.

**Table 2.** Performance of machine learning models on the test set.

Model	Accuracy	Precision	Recall	*F*_1_-score	AUC^a^
RF^b^	0.8483	0.8258	0.8258	0.8258	0.9261
XGBoost^c^	0.8186	0.7895	0.7955	0.7925	0.8742
SVM^d^	0.7585	0.7140	0.7424	0.7280	0.8103
LR^e^	0.6735	0.6130	0.6780	0.6439	0.7293

a AUC: area under the curve.

b RF: random forest.

c XGBoost: extreme gradient boosting.

d SVM: support vector machine.

e LR: logistic regression.

### Ranking the Importance of Features Across Different Variables

**Figure 1.**
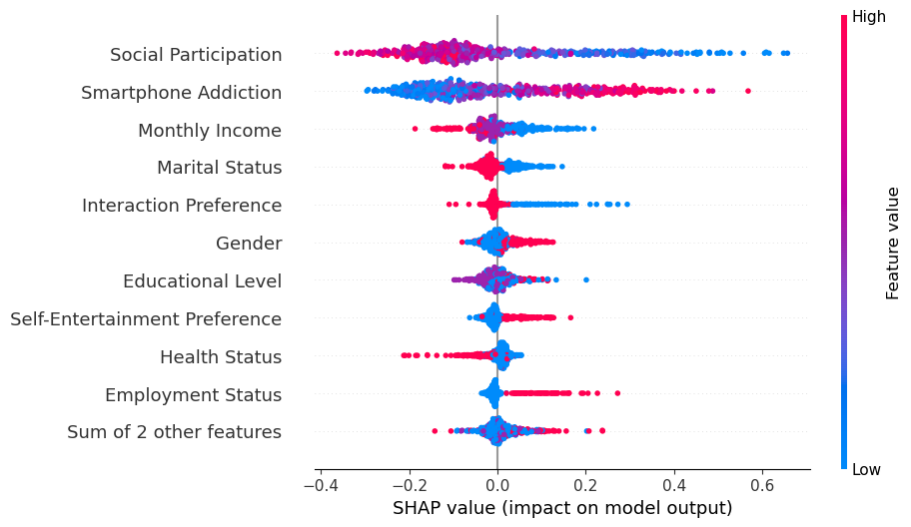
Predictors of depression. SHAP: Shapley additive explanations.

As shown in Table 3, the most influential predictors of depression, in descending order of importance, were social participation, smartphone addiction, monthly income, marital status, interaction preference (ie, the use of interactive functions), education level, gender, health status, self-entertainment preference (ie, the use of self-entertainment functions), and age, with social participation and smartphone addiction being the most salient factors. Figure 1 presents the Shapley additive explanations algorithm values, indicating that social participation had the strongest negative association with depression, whereas higher levels of participation were associated with a significantly lower probability of depression. Smartphone addiction was positively associated with depression. Higher income and being married were associated with a lower risk of depression, and use of interactive functions preference was also associated with reduced risk. Educational attainment showed a relatively modest association with a risk of depression. Female sex, poorer health status, a stronger preference for self-entertainment functions, and older age were each associated with a slight increase in depression risk. Because fsQCA typically examines 4 to 8 antecedent conditions, the top 7 predictors—social participation, smartphone addiction, monthly income, marital status, use of interactive functions, education, and gender—were selected as key conditions for configurational analysis of late-life depression.

**Table 3.** Top 10 features ranked by Shapley additive explanations (SHAP) algorithm values.

Importance rank	Feature	SHAP value
1	Social participation	0.1613
2	Smartphone addiction	0.1441
3	Monthly income	0.0421
4	Marital status	0.0272
5	Preference for interactive functions	0.0230
6	Educational level	0.0211
7	Gender	0.0201
8	Health status	0.0199
9	Preference for self-entertainment functions	0.0194
10	Age	0.0192

### FsQCA Analysis

This study used fsQCA 4.0 software to convert antecedent and outcome variables into fuzzy-set membership scores using the direct calibration method. Following the literature in FsQCA analysis, the 95th, 50th, and 5th percentiles were set as the 3 anchor points for full membership, the crossover point, and full nonmembership, respectively [29,30]. To address the issue that cases with a fuzzy membership score of exactly 0.5 cannot be included in the analysis, the method proposed by Fiss [31] was followed, replacing the 0.5 value with 0.501. Table 4 presents the results of the variable calibration.

**Table 4.** Calibration analysis.

Variables	Anchor points^a^		
	Full nonmembership	Crossover point	Full membership
Condition variables			
Social participation	–1.722	0.662	1.790
Smartphone addiction	–1.224	0.018	1.624
Monthly income	0	1	2
Marital status	0	1	1
Preference for interactive functions	0	1	1
Education level	0	1	2
Gender	0	0	1
Outcome variable			
Depression	0	0	2

a Anchor points were calibrated using the 5th percentile (full nonmembership), 50th percentile (crossover), and 95th percentile (full membership).

To test the first hypothesis, which posited that high smartphone addiction is a necessary condition for high depression, a necessity analysis was conducted. As shown in Table 5, the consistency score for smartphone addiction was 0.991, exceeding the commonly used threshold of 0.9. This suggests that nearly all cases of high depression also exhibited high smartphone addiction. Therefore, the first hypothesis is strongly supported. The consistency scores for the remaining variables were all below 0.9, indicating that none met the threshold for depression in individual cases. The combined effects of multiple variables warrant further examination using a configurational approach.

**Table 5.** Fuzzy-set qualitative comparative analysis necessity analysis.

Condition variables	Consistency	Coverage
Social participation		
Yes	0.490	0.887
No	0.824	0.841
Smartphone addiction		
Yes	0.991	0.974
No	0.508	0.985
Monthly income		
Yes	0.675	0.757
No	0.616	0.744
Marital status		
Married	0.030	0.789
Unmarried	0.634	0.665
Use of interactive functions		
Yes	0.013	0.918
No	0.994	0.658
Education		
Any formal education	0.560	0.780
No formal education	0.680	0.707
Gender		
Male	0.745	0.746
Female	0.534	0.639

The analysis identified 3 distinct configurations (Table 6), which collectively achieved a high overall fsQCA configurational (sufficiency) solution of 0.956. The first configuration, termed the digitally disconnected and vulnerable profile, is characterized by high smartphone addiction, low interaction preference, lower educational attainment, and male gender. The raw coverage was 0.499, indicating that approximately 49.9% of cases of depression were represented by this configuration. This finding supports the hypothesized risk pattern in contexts where structural constraints may amplify behavioral vulnerabilities. The second and third configurations highlight more complex, paradox-driven mechanisms. The socially withdrawn profile with greater socioeconomic resources was characterized by high smartphone addiction, low social participation, and no use of interaction functions, despite high educational attainment and high income. This configuration demonstrated a raw coverage of 0.560. This supports the hypothesis that greater socioeconomic resources may not necessarily protect against depression when digital use is maladaptive, suggesting a potential paradox among socioeconomically advantaged older adults. The third configuration, the emotionally isolated and digitally dependent profile, was specifically characterized by unmarried men with high smartphone addiction and low interaction preference, even when overall social participation appeared adequate. The raw coverage was 0.462. Collectively, these 3 configurations illustrated how smartphone addiction, in combination with specific socioeconomic and marital contexts, was associated with late-life depression.

The analysis revealed an additional sufficient configuration that was not prespecified in the hypotheses. This emergent configuration, termed the emotionally isolated and digitally dependent profile, demonstrated a consistency of 0.910 and unique coverage of 0.011. It was characterized by unmarried older men who, despite relatively high social participation, exhibited high smartphone addiction and no use of interaction functions. The identification of this configuration is an important finding. Whereas the initial second and third hypotheses focused on vulnerabilities associated with limited socioeconomic resources or low social participation, this configuration suggested a distinct pattern associated with relational and gender-related vulnerabilities. The theoretical implications of this unanticipated finding are discussed further in the Discussion section. The robustness of the solution was assessed by (1) raising the consistency threshold from 0.80 to 0.85, (2) tightening the proportional reduction in inconsistency cutoff, and (3) modifying the case frequency threshold. The core configurations for depression remained substantively unchanged, confirming the stability of the solution.

**Table 6.** Configurations associated with depression.^a^^,^^b^

Variables and metrics	Depression		
	Configuration 1	Configuration 2	Configuration 3
Condition variables			
Social participation		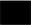 ^c^	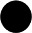 ^d^
Marital status	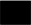		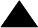 ^e^
Interaction preference		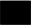	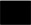
Education	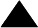	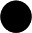	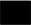
Gender	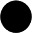		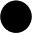
Monthly income	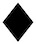 ^f^	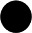	
Smartphone addiction	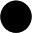	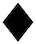	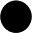
Solution metrics			
Consistency	0.928	0.978	0.910
Raw coverage	0.499	0.560	0.462
Unique coverage	0.160	0.043	0.011

a Overall solution consistency was 0.956.

b Overall solution coverage was 0.243.

c Indicates the absence of core conditions.

d Indicates the presence of core conditions.

e Indicates the absence of peripheral conditions.

f Indicates the presence of peripheral conditions.

## Discussion

### Configurational Patterns of Late-Life Depression in the Digital Context

This study confirmed smartphone addiction as a foundational vulnerability and necessary condition for late-life depression. However, this individual-level risk factor appeared to operate through specific configurational pathways rather than in isolation. Thus, this study synthesized the findings into a dual-mechanism integrated framework of depression caused by digital technology to clarify heterogeneity in etiological patterns across different groups.

The first mechanism, structural exclusion and deficit, reflects a resource-deficit pattern of depression primarily observed among older men with low educational attainment. This pattern may be shaped by digital inequality and gender-related social norms [32]. Limited educational attainment may constrain digital literacy, contributing to a greater reliance on smartphone use to engage in passive entertainment rather than socially connective functions. In contrast to women, who often maintain broader kin-keeping networks, older men may rely more heavily on spouse-centered networks. Following widowhood or social isolation, no use of interaction functions and lower digital literacy may hinder the formation of online connections, while fewer kin-keeping practices may reduce social buffers against loneliness. These intersecting factors are associated with an elevated risk of depression in this subgroup [33,34]. In this context, smartphones may not function as bridges to social integration and instead may reinforce existing structural marginalization.

The second mechanism, agentic maladaptation and relational void, reflects a maladaptation-based pattern of depression observed among individuals with greater socioeconomic resources and unmarried men. For socioeconomically advantaged individuals, material resources such as income and education may not provide effective psychological protection in the absence of socially meaningful connections. Consistent with SST, passive digital consumption without interpersonal interaction cannot substitute for emotionally meaningful engagement, potentially leading to material abundance coexisting with emotional deprivation [35]. Among unmarried men, the absence of a primary attachment figure may create a unique emotional void. Even when overall social participation seems adequate, reliance on noninteractive smartphone use may represent an attempt to use technology for unmet emotional needs. In this configuration, depression was associated not with limited capability or access but with patterns of digital engagement that may reflect efforts to cope with offline relational deficits through compulsive digital escapism [36].

### Theoretical and Practical Implications

This study contributes to DIT in two ways. First, it refines DIT by identifying gendered patterns among men with lower educational attainment. Second, it extends SST by specifying contextual conditions under which unmet social connection needs are associated with depression, even among groups with greater socioeconomic resources, highlighting the importance of meaningful engagement for well-being.

From a practical perspective, these findings suggest that interventions should be targeted and configuration-specific rather than uniform. For digitally disconnected older men at elevated risk, intervention strategies may extend beyond basic technical support to strengthen digital family connections. Community-based workshops (eg, grandparent-grandchild connection programs) can teach functional skills such as video calling and photo sharing, transforming passive screen time into active intergenerational engagement. Peer-led interest groups may also promote social engagement.

For socioeconomically advantaged but socially isolated older adults, greater resources may not prevent depression associated with a diminished sense of purpose. Meaning-oriented engagement, such as recruiting older adults as online mentors, may shift their role from passive consumer to active contributor. Among unmarried older men, the combination of unmarried status and high screen time may warrant proactive screening. Programs such as digital workshops for older men could create low-pressure online spaces that foster weak-tie networks, reduce loneliness, and decrease emotional dependency on smartphones. Finally, given that smartphone addiction was identified as a necessary condition across depressive profiles, technology developers should consider incorporating age-sensitive design principles and responsible algorithm practices to support digital well-being.

These findings should be considered alongside several limitations. First, the cross-sectional design and nonprobability sampling from Guangzhou limit causal inference and generalizability. Longitudinal studies are needed to clarify causality and reduce potential measurement overlap between smartphone addiction and depression. Second, the measure of social participation did not distinguish between online and offline engagement, which may obscure their distinct associations with mental health. Future research should use instruments that differentiate these modalities. Third, smartphone addiction in older adults may require more nuanced assessment. Behaviors resembling addiction may reflect limited digital literacy rather than true dependency. Mixed-methods studies could help distinguish between compulsive use and unskilled engagement. Fourth, the high internal consistency (Cronbach α=0.968) of the Mobile Phone Addiction Index in this sample suggests it may be perceived as a unidimensional construct by older adults; further validation of its factor structure in this population is warranted. Finally, this study did not account for medication use, a potential confounder. Future research should incorporate medication-related variables to better isolate the association between digital engagement and depressive symptoms.

### Conclusion

This study suggests that late-life depression and the increasing use of smartphones is not a uniform condition but reflects complex interactions. Smartphone addiction was consistently associated with an increased risk of depression and appeared to operate through 2 distinct mechanisms: structural exclusion and maladaptive engagement. The former was characterized by resource deficits among vulnerable men, whereas the latter was associated with relational isolation among individuals with greater socioeconomic resources or limited social integration. These results support the use of a configurational approach rather than single-factor explanations to promote healthy aging in an increasingly digital society.

## Abbreviations

DIT: digital inequality theory
fsQCA: fuzzy-set qualitative comparative analysis
RF: random forest
SST: socioemotional selectivity theory
